# Transforming growth factor-β-induced secretion of extracellular vesicles from oral cancer cells evokes endothelial barrier instability via endothelial-mesenchymal transition

**DOI:** 10.1186/s41232-022-00225-7

**Published:** 2022-09-04

**Authors:** Miho Kobayashi, Kashio Fujiwara, Kazuki Takahashi, Yusuke Yoshioka, Takahiro Ochiya, Katarzyna A. Podyma-Inoue, Tetsuro Watabe

**Affiliations:** 1 Department of Biochemistry, Graduate School of Medical and Dental Sciences, Tokyo Medical and Dental University (TMDU), 1-5-45 Yushima, Bunkyo-ku, Tokyo, 113-8549 Japan; 2 Institute of Industrial Science, The University of Tokyo, 4-6-1 Komaba, Meguro-ku, Tokyo, 153-8505 Japan; 3 Department of Molecular and Cellular Medicine, Institute of Medical Science, Tokyo Medical University, 6-7-1 Nishishinjuku, Tokyo, 160-0023 Japan

**Keywords:** Extracellular vesicles, Epithelial-mesenchymal transition, Endothelial-mesenchymal transition, Transforming growth factor-β, Endothelial barrier, Pre-metastatic niches

## Abstract

**Background:**

During metastasis, cancer cells undergo epithelial-mesenchymal transition (EMT) in response to transforming growth factor-β (TGF-β), which is abundant in the tumor microenvironment, and acquire invasive and metastatic potentials. Metastasis to distant organs requires intravascular invasion and extravasation of cancer cells, which is accompanied by the disruption of the adhesion between vascular endothelial cells. Cancer cell-derived extracellular vesicles (EVs) have been suggested to induce the destabilization of normal blood vessels at the metastatic sites. However, the roles of EVs secreted from cancer cells that have undergone EMT in the destabilization of blood vessels remain to be elucidated. In the present study, we characterized EVs secreted by oral cancer cells undergoing TGF-β-induced EMT and elucidated their effects on the characteristics of vascular endothelial cells.

**Methods:**

Induction of EMT by TGF-β in human oral cancer cells was assessed using quantitative RT-PCR (qRT-PCR) and immunocytochemistry. Oral cancer cell-derived EVs were isolated from the conditioned media of oral cancer cells that were treated with or without TGF-β using ultracentrifugation, and characterized using nanoparticle tracking analysis and immunoblotting. The effects of EVs on human umbilical artery endothelial cells were examined by qRT-PCR, cellular staining, and permeability assay. The significant differences between means were determined using a *t*-test or one-way analysis of variance with Tukey’s multiple comparisons test.

**Results:**

Oral cancer cells underwent EMT in response to TGF-β as revealed by changes in the expression of epithelial and mesenchymal cell markers at both the RNA and protein levels. Oral cancer cells treated with TGF-β showed increased EV production and altered EV composition when compared with untreated cells. The EVs that originated from cells that underwent EMT by TGF-β induced endothelial-mesenchymal transition, which was characterized by the decreased and increased expression of endothelial and mesenchymal cell markers, respectively. EVs derived from oral cancer cells also induced intercellular gap formation which led to the loss of endothelial cell barrier stability.

**Conclusions:**

EVs released from oral cancer cells that underwent TGF-β-induced EMT target endothelial cells to induce vascular destabilization. Detailed characterization of oral cancer-derived EVs and factors responsible for EV-mediated vascular instability will lead to the development of agents targeting metastasis.

**Supplementary Information:**

The online version contains supplementary material available at 10.1186/s41232-022-00225-7.

## Background

Interaction between cancer cells and endothelial, immune, or stromal cells present in the tumor microenvironment (TME) plays an important role in cancer progression [1]. Blood vessels also play an important role in cancer progression. They facilitate tumor growth by supplying nutrients and serve as migration routes during the metastasis of cancer cells. During the intravasation of cancer cells into blood vessels and extravasation of metastasizing cancer cells, the adhesion between vascular endothelial cells is altered, leading to the dysfunction of the endothelial cell barrier and the instability of blood vessels [2]. Therefore, understanding the mechanism controlling distant metastasis and identifying the factors regulating this process are important for developing effective therapeutic strategies.

Transforming growth factor-β (TGF-β) is a multifunctional cytokine abundant in the TME and a poor prognostic factor in many cancers, including oral cancer [3]. TGF-β promotes cancer progression by affecting various components of the TME, including cancer cells, blood vessels, immune cells, and cancer-associated fibroblasts [4]. TGF-β signaling acts through its serine-threonine kinase type receptors (type I and type II receptors). The binding of TGF-β to receptors activates the TGF-β signaling pathway, resulting in the phosphorylation of the intracellular signaling components Smad2/3 [5]. Phosphorylated Smad2/3 form a complex with Smad4 and translocate to the nucleus, leading to the expression of various direct target genes [6, 7]. Activation of TGF-β signaling can induce various cellular responses, including epithelial-mesenchymal transition (EMT). EMT is a process during which epithelial cancer cells lose their epithelial features and acquire mesenchymal phenotypes, leading to enhanced cell motility and invasiveness [5]. EMT is characterized by the downregulated expression of epithelial cell markers, E-cadherin, and claudin-1 and the upregulated expression of mesenchymal markers, smooth muscle protein 22α (SM22α), and vimentin.

TGF-β also acts on vascular endothelial cells in the TME and induces endothelial-mesenchymal transition (EndoMT) [8, 9]. Similar to EMT, EndoMT is characterized by the loss of endothelial cell integrity and decreased expression of endothelial cell markers, vascular endothelial growth factor receptor 2 (VEGFR2), VE-cadherin, and Tie-2, which is accompanied by increased expression of mesenchymal cell markers, SM22α, α-smooth muscle actin (αSMA), and matrix metalloproteinase 2 (MMP2). It has been reported that the altered expression of these genes during EndoMT is mediated by Slug and Snail, known as EndoMT-related transcription factors [8, 9]. Under normal physiological conditions, EndoMT occurs during embryogenesis and cardiac valve formation, but EndoMT is also known to destabilize vascular structures and increase vascular permeability [10]. Therefore, EndoMT has been suggested to facilitate intravasation and extravasation of cancer cells from the blood vessels and promote cancer metastasis.

Extracellular vesicles (EVs) are important mediators of extracellular signaling through direct interactions with cell surface receptors, activation of signaling pathways or direct transfer of biologically active molecules into recipient cells [11, 12]. Particularly, small EVs with a diameter of approximately 100 nm secreted by cancer cells have drawn much attention. EVs carry various cytoplasmic- and membrane-bound proteins and nucleic acids. The cargo of EVs can be transferred to cells residing in the vicinity, and in distant organs. The uptake of EVs can reprogram recipient cells leading to changes in their phenotypes and functions [13]. Multiple groups have reported that EVs released from cancer cells promote tumor development by inducing EMT, which increases the invasive and migratory potentials of cancer cells [14–17].

Recent lines of evidence pointed important roles of EVs in vascular biology. EVs released by cancer cells have been reported to enhance the adhesion between cancer cells and vascular endothelial cells by affecting normal blood vessels [18, 19]. In addition, EVs released from breast cancer cells were shown to act on normal vascular endothelial cells and induce distant metastasis through increased vascular permeability [20]. Another report suggested that cancer cell-derived EVs may induce the formation of pre-metastatic niches (PMNs) in normal blood vessels at metastatic sites, contributing to distant metastasis [21]. However, the effects of cancer cell-derived EVs on EndoMT in normal vascular endothelial cells remain unclear. In the present study, we characterized EVs secreted by oral cancer cells undergoing EMT and elucidated their effects on normal vascular endothelial cells. Our data revealed that oral cancer cells undergoing TGF-β-induced EMT released EVs, which triggered the changes in endothelial cells by inducing EndoMT.

## Methods

### Cell culture and reagents

Human oral squamous cell carcinoma (OSCC) cell lines: HSC-4 and SAS were obtained from RIKEN BioResource Center Cell Bank. The cells were maintained in Dulbecco’s modified Eagle’s medium (DMEM; Nacalai Tesque, #08458-16) supplemented with 10% fetal bovine serum (FBS, Gibco, #11573397). For induction of EMT, OSCC cells were stimulated with 3 ng/ml TGF-β1 (PeproTech, #100-21C). Human umbilical artery endothelial cells (HUAECs) were purchased from Cell System and maintained in EGM-2 MV Bullet Kit (Lonza, CC-3202).

### EV isolation

OSCC cells were cultured with or without TGF-β1 for 72 h. The medium was then replaced with serum-free Advanced DMEM (Gibco, #12491015), and the cells were incubated for additional 48 h to allow accumulation of EVs in the conditioned medium. The conditioned media were then collected, pre-cleared by centrifugation at 2000 × g at 4°C for 10 min, and passed through a 0.22-μm pore filter (IWAKI, #8020-250). The collected culture supernatant was then subjected to ultracentrifugation at 150,000 × g at 4°C for 90 min (Beckman Optima XE-90, rotor: SW28 lot #S/N 20U12096) to isolate EVs. EVs were washed with phosphate-buffered saline (PBS) followed by an additional ultracentrifugation step and used in further experiments. Purified EVs (final concentration: 1 × 10^11^ particles/ml) were used to stimulate HUAECs for 72 h, followed by quantitative RT-PCR (qRT-PCR) and immunocytochemical analysis as described in later sections.

### Quantification and characterization of EVs using Nanoparticle Tracking Analysis (NTA)

The number and size of isolated EVs were determined by NTA using the NanoSight system (NanoSight NTA3.4, blue laser: 405 nm) as reported previously [22]. Briefly, collected EVs were diluted 1000-fold with PBS, and the light scattered from particles was recorded for 60 s (camera level 13) followed by analysis with NTA software (NTA3.2) to calculate size and concentration of EVs in each sample.

### qRT-PCR analysis

Total RNA was purified with RNeasy Plus Mini kit (Qiagen, #74134), and cDNA was synthesized using PrimeScript II 1st strand cDNA Synthesis Kit (TaKaRa Bio, #6210A). The qRT-PCR was performed with the ABI StepOnePlus system (Applied Biosystems) using gene-specific primers and Fast Start Universal SYBR Green Master (Roche, #04913914001). The relative standard curve method was used to determine the relative expression of target genes [23]. All expression data were normalized to the expression of β-actin. The genes and corresponding primer sequences are listed in Supplementary Table 1.

### Immunoblotting

EVs (1.4 × 10^10^ particles) and cell lysates (20 μg of total proteins) were separated in polyacrylamide gel (TGX FastCast Acrylamide Kit, 7.5%, Bio-Rad, #1610171) (TGX FastCast Acrylamide Kit, 12%, Bio-Rad, #1610175) electrophoresis and transferred onto a PVDF membrane (Millipore, #IPVH00010). The antibodies to EV markers: Alix (Sigma-Aldrich, #SAB4200477), TSG101 (Abcam, #ab30871), and CD63 (BD Bioscience, #556019), and antibody to Golgi marker: Golgi Reassembly Stacking Protein 1 (GORASP1) (Sigma-Aldrich, #SAB4100063) were used to detect separated proteins. The membranes were then incubated with anti-rabbit IgG-HRP (Cell Signaling Technology, #7074) or anti-mouse IgG-HRP (Cell Signaling Technology, #7076). Binding of specific antibodies was detected with Western chemiluminescent HRP substrate (Millipore, #WBKLS0500), visualized, and quantified with FUSION SOLO S (Vilber Lourmat). The intensity level of the band corresponding to each molecule was normalized by its intensity in the corresponding cell lysate.

### Immunocytochemical analysis

HSC-4 cells cultured on cover glass (Matsunami, #5001) were washed with PBS, fixed with ice-cold acetone/methanol (1:1), blocked with 2% FBS for 40 min, and incubated with primary antibodies to E-cadherin (Cell Signaling Technology, #14472S) and vimentin (Abcam, #ab2547). The molecules were then visualized with secondary antibodies: Alexa Fluor-488 anti-mouse IgG (Invitrogen, #A21202) and Alexa Fluor-647 anti-rabbit IgG (Invitrogen, #A31573). Nuclei were stained with Hoechst 33342 (Invitrogen, #H1399). HUAEC monolayers cultured on collagen type I-coated cover glasses (IWAKI, #11-0071) were washed with PBS, fixed with 4% paraformaldehyde solution for 15 min, and permeabilized with 0.1% Triton X-100 for 15 min. The samples were then blocked with 2% FBS for 40 min and incubated with anti-VE-cadherin (Sigma-Aldrich, #MABT129) and SM22α (Abcam, #GR3321006-1) primary antibodies, followed by incubation with secondary antibodies: Alexa Fluor-488 anti-mouse IgG and Alexa Fluor-647 anti-rabbit IgG. Hoechst 33342 was used to visualize nuclei. Stained HSC-4 cells or HUAECs were mounted and observed with a Keyence BZ-X710 fluorescence microscope or a Leica TCS SP8 confocal laser scanning microscope. Quantitative analysis of obtained images (five fields of view from at least four independent samples) was done with Fiji (ImageJ).

### Endothelial monolayer gap formation analysis

HUAEC monolayers were cultured on collagen type I-coated cover glass and treated with EVs (Control-EVs or TGF-β-EVs) for 72 h. Gap formation analysis was done as reported previously [24] using immunocytochemical images of HUAECs representing staining for VE-cadherin and nuclei. In brief, the gap area was defined as the area not occupied by HUAECs and thus negative for both VE-cadherin and nucleus staining. At least five fields of view from at least four independent samples were analyzed using Fiji (ImageJ). The gap formation was expressed as the percentage of formed gap in the total area of each image.

### Endothelial monolayer permeability assay

HUAEC monolayers were cultured on transwell culture insert (1 μm pore, FALCON, #353104) and treated with EVs (Control-EVs or TGF-β-EVs) for 72 h. Permeability assay was performed previously described [25–27]. Briefly, the 10 μl of 100 μg/ml FITC-dextran (70 kDa) (Merck, #46945) in the EGM-2MV medium were added into the upper well containing HUAEC monolayer followed by incubation for 30 min. To quantify the HUAEC monolayer permeability, the fluorescence intensity of FITC-dextran leaking into the lower well was measured with FLUOstar OPTIMA-6 (BMG Labtech). The obtained values were averaged and were considered as representative of leakage in HUAEC monolayers related to increased permeability to 70-kDa molecules.

### Statistics

Values are presented as mean ± standard deviation (SD), and significant differences between means were determined with two-tailed Student’s *t*-test or ordinary one-way analysis of variance (ANOVA) with Tukey’s multiple comparisons test using Prism 9 software version 9.1.2 (GraphPad). Differences between means were considered statistically significant at *P* < 0.05.

## Results

### TGF-β induces EMT in human oral cancer cells

**Fig. 1:**
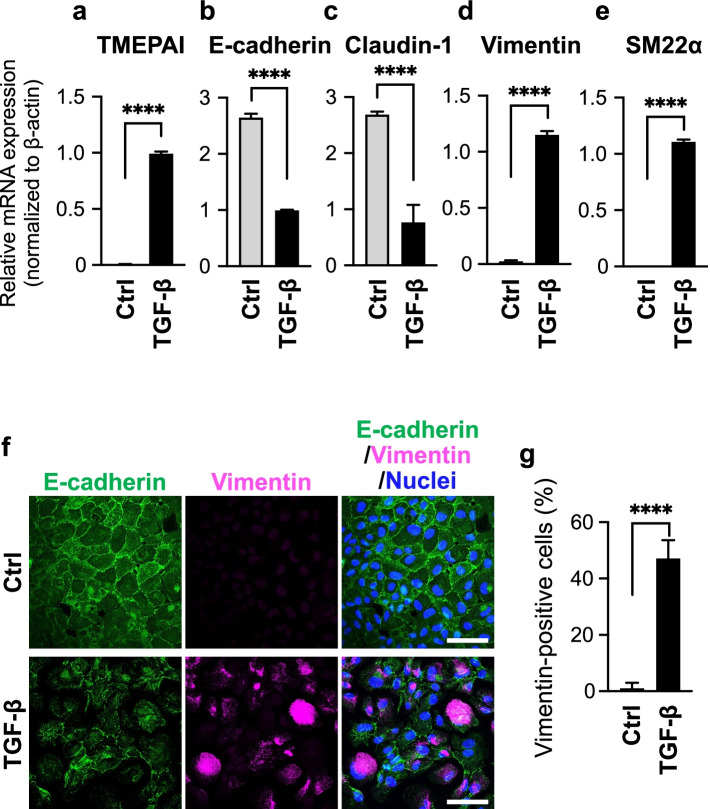
TGF-β induces EMT in oral cancer cells. HSC-4 cells were stimulated without (Ctrl) or with 3 ng/ml TGF-β for 72 h. **a–e** Total RNA was analyzed by qRT-PCR using each specific primers for TMEPAI (**a**), E-cadherin (**b**), claudin-1 (**c**), vimentin (**d**), or SM22α (**e**). Data represent fold changes relative to β-actin. **f** Confocal images for the expression of E-cadherin and vimentin in HSC-4 cells. Cells were fixed and stained with anti-E-cadherin (green), anti-vimentin (magenta), and Hoechst33342 (nuclei: blue). Scale bars, 50 μm. **g** Quantification of vimentin-positive cells. All data are represented as the mean ± SD from three independent experiments. ****P < 0.0001 by two-tailed Student’s *t*-test

TGF-β can induce EMT in epithelial oral cancer cells and facilitate cancer progression by increasing the metastatic potential of oral cancer cells [3]. The number and characteristics of EVs released by cancer cells were reported to possibly change depending on the metastatic properties of cancer cells [27]. Therefore, the human OSCC cell line, HSC-4 was cultured in the presence of TGF-β for 72 h, and EMT induction was evaluated by qRT-PCR and immunocytochemistry. TGF-β signaling was activated in HSC-4 cells, as revealed by the upregulated expression of transmembrane prostate androgen-induced protein (TMEPAI), a direct target gene of TGF-β signaling (Fig. 1a). The qRT-PCR analysis revealed that the treatment of HSC-4 cells with TGF-β reduced the expression of the epithelial cell markers, E-cadherin (Fig. 1b) and claudin-1 (Fig. 1c). These changes were accompanied by the upregulated expression of the mesenchymal cell markers, vimentin (Fig. 1d) and SM22α (Fig. 1e), confirming the previous findings [3]. HSC-4 cells cultured in the presence of TGF-β lost the contacts between adjacent cells (hereafter termed “cell-cell contacts”), which is characteristic for epithelial cells. HSC-4 cells treated with TGF-β also showed the reduced E-cadherin staining intensity (Fig. 1f, lower panels) compared with the untreated, control cells (Fig. 1f, upper panels). The localization of E-cadherin in TGF-β-treated cells was more cytoplasmic than that in control cells. The changes in E-cadherin staining were also accompanied by a significant increase in the number of vimentin-positive cells in the TGF-β-treated cells compared with that in the control cells (Fig. 1f, g), confirming that HSC-4 cells undergo EMT upon stimulation with TGF-β.

### TGF-β-treated oral cancer cells increase the release of EVs

**Fig. 2:**
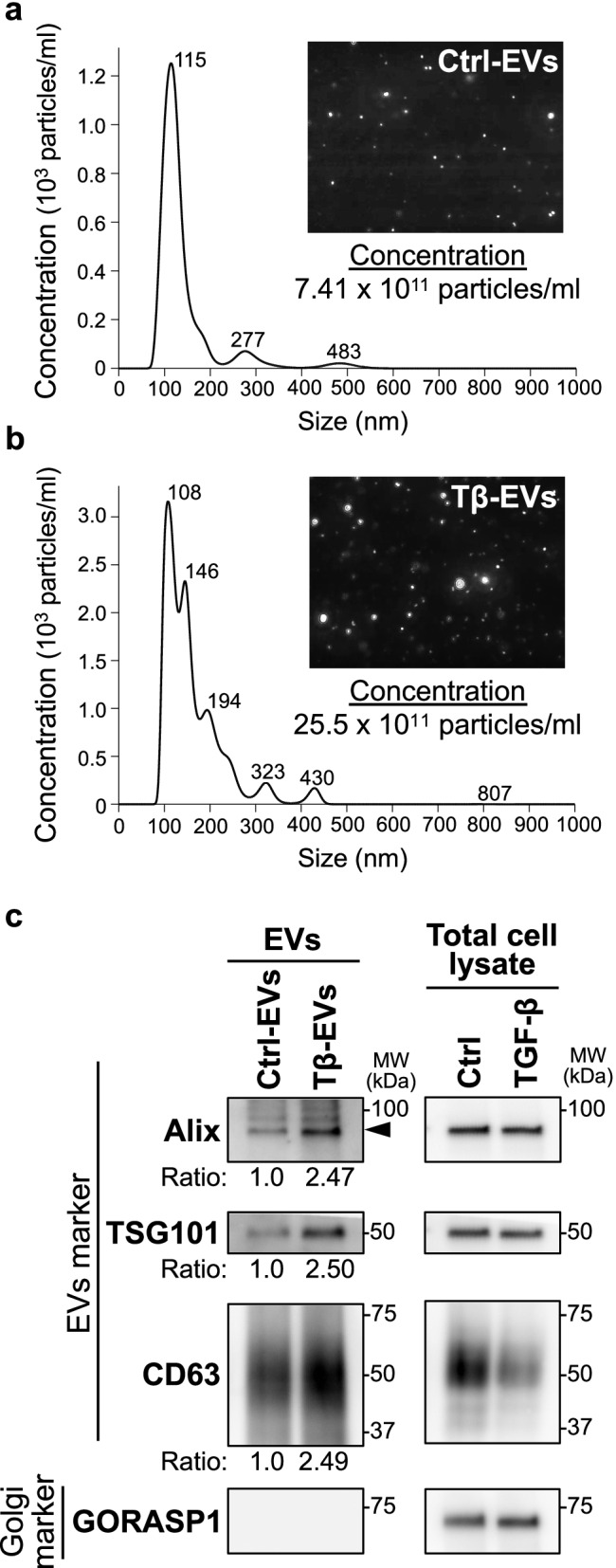
TGF-β increases the release of EVs from oral cancer cells. **a**, **b** Nanoparticle tracking analysis of EVs from the conditioned medium of HSC-4 cells treated without (**a** Ctrl-EVs) or with TGF-β (**b** Tβ-EVs) using the NanoSight system. The inset: NanoSight capture images show particles isolated from the same volume of each conditioned medium. **c** Immunoblotting analysis of total cell lysate of HSC-4 cells stimulated without (Ctrl) or with 3 ng/ml TGF-β (TGF-β) for 72 h, and the respective EV fraction (Ctrl-EVs and Tβ-EVs). Samples derived from the same number of particles were loaded onto each lane. Antibodies against EV markers (Alix, TSG101, and CD63) and Golgi apparatus marker (GORASP1) were used for analysis. Cropped blot images are shown. See Supplementary Information for the original full-length blot images. The intensity of each band was then quantified. The relative value was normalized to that of the cell lysate and is shown as ratio

More metastatic cancer cells were shown to release more EVs than their non-metastatic counterparts [28]. Therefore, in the next step, we isolated EVs secreted by HSC-4 cells treated with or without TGF-β (hereafter termed “TGF-β-EVs” and “Control-EVs,” respectively) and subjected them to quantitative and qualitative analyses using NanoSight and immunoblotting. Nanoparticle analysis using NanoSight revealed that cells treated without and with TGF-β released EVs (Fig. 2a, b). Majority of these particles corresponded to the EVs with the size of approximately 100 nm (Fig. 2a, b). Notably, analysis using NanoSight revealed that cells treated with TGF-β released approximately 3-times more EVs than control cells cultured without TGF-β (Fig. 2a, b). The identity of EVs was further confirmed by immunoblotting analysis with antibodies to proteins residing in EVs: Alix, tumor susceptibility gene 101 protein (TSG101), and CD63 (Fig. 2c, left panels) and lack of reactivity to the Golgi marker, Golgi reassembly-stacking protein 1 (GORASP1) antibodies (Fig. 2c left panel). Notably, qualitative analysis of the intensity of bands corresponding to EV markers also revealed the enrichment of Alix, TSG101, and CD63 in EVs derived from TGF-β-stimulated HSC-4 cells when compared with EVs isolated from untreated cells (Fig. 2c). These results suggest that oral cancer cells, which underwent TGF-β-induced EMT, not only increased the number of secreted EVs, but also altered their cargo which may be important regarding the later effects exerted by EVs.

### EVs derived from oral cancer cells which underwent TGF-β-induced EMT induce EndoMT in human endothelial cells

**Fig. 3:**
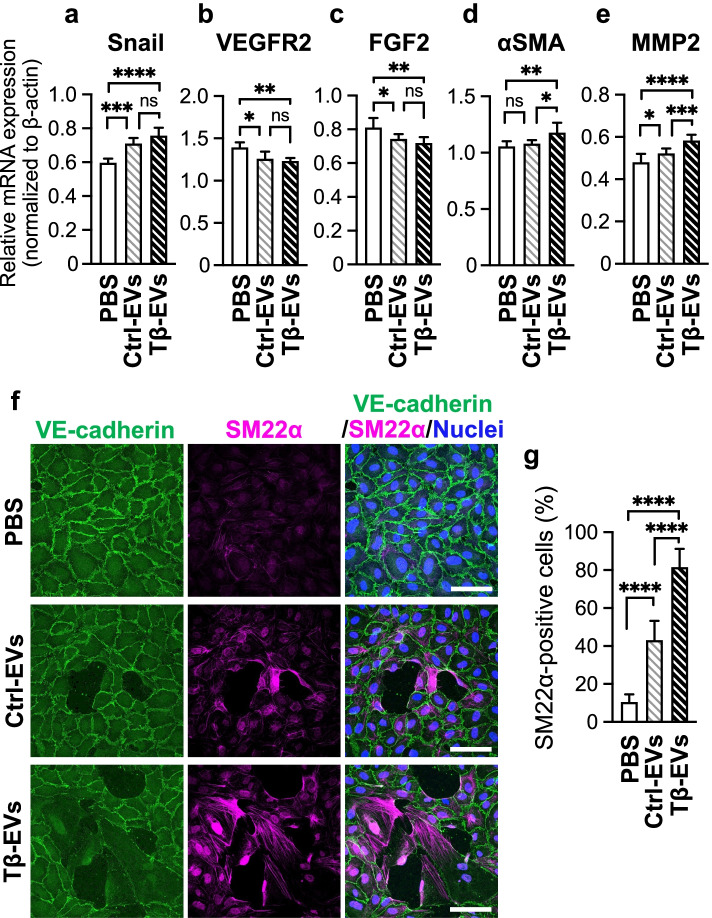
Oral cancer cell-derived EVs induce EndoMT in vascular endothelial cells. **a**–**e** HUAEC monolayers were treated with vehicle (PBS) or EVs (Ctrl-EVs or Tβ-EVs) for 72 h. **a**–**e** The expression of Snail (**a**), VEGFR2 (**b**), FGF2 (**c**), SM22α (**d**), or MMP2 (**e**) was analyzed by qRT-PCR. Data represent fold changes relative to β-actin. **f** Confocal images showing both the localization of VE-cadherin and the expression of SM22α in HUAECs. Cells were fixed and stained with anti-VE-cadherin (green) and anti-SM22α (magenta) antibodies. Nuclei were stained with Hoechst33342 (blue). Scale bars, 50 μm. **g** Quantification of SM22α-positive cells. All data are shown as the mean ± SD from three independent experiments. *P < 0.05, **P < 0.01, ***P < 0.001, ****P < 0.0001 by ordinary one-way ANOVA with Tukey’s multiple comparisons test; ns, not significant

TGF-β stimulation affected the quantity and quality of EVs released from oral cancer cells; therefore, we examined the effect of EVs on cells residing in the TME. It has been suggested that EVs released from cancer cells affect normal vascular endothelial cells [18, 19]. Therefore, we stimulated HUAECs with EVs isolated from untreated control oral cancer cells (Control-EVs) and oral cancer cells treated with TGF-β (TGF-β-EVs) and determined the changes induced upon incubation with EVs by using qRT-PCR and immunocytochemistry. The incubation of HUAECs with Control-EVs and TGF-β-EVs slightly increased the expression of EndoMT-related transcription factors, Snail (Fig. 3a) and Slug (Supplementary Fig. 1a), compared with PBS-treated cells. The expressions of endothelial cell markers, VEGFR2 (Fig. 3b) and Tie2 (Supplementary Fig. 1b), as well as fibroblast growth factor-2 (FGF2, Fig. 3c), which is known to be indispensable for maintaining the endothelial characteristics [29], were decreased by treatment with both Control-EVs and TGF-β-EVs. However, no significant difference was observed between the effects exerted by Control-EVs and TGF-β-EVs on the expression of each marker (Fig. 3a–c). By contrast, TGF-β-EVs significantly increased the expression of mesenchymal cell markers, αSMA, MMP2 (Fig. 3d, e), and SM22α (Supplementary Fig. 1c), compared with Control-EVs. Notably, neither type of EV increased the expression of TMEPAI (Supplementary Fig. 1d), suggesting that oral cancer cell-derived EVs induced EndoMT without activation of TGF-β signaling.

Furthermore, we performed immunocytochemical analysis using antibodies against the endothelial cell marker, VE-cadherin, and the mesenchymal marker, SM22α. HUAEC monolayers were incubated with either vehicle (PBS) or EVs (Control-EVs or TGF-β-EVs). Both Control-EVs and TGF-β-EVs increased the number of SM22α-positive cells, with a much stronger effect exerted by TGF-β-EVs than Control-EVs (Fig. 3f, g). These results were confirmed using another OSCC cell line, SAS which has been reported to undergo EMT in response to TGF-β [3] (Supplementary Fig. 2), suggesting that TGF-β-EVs induce EndoMT in human endothelial cells more effectively than Control-EVs.

### EVs derived from oral cancer cells which underwent TGF-β-induced EMT disrupt endothelial cell barrier

Blood vessels form a closed system that exhibits integrity of the junction between adjacent cells (hereafter termed “cell-cell adhesion”), which is important for stabilization of the barrier function to prevent blood leakage. This characteristic can be achieved by forming strong cell-cell adhesion between vascular endothelial cells; therefore, any disruption of cell-cell adhesion will result in vascular destabilization [30, 31]. In HUAECs incubated with PBS, VE-cadherin accumulated at cell-cell contact sites, indicating the formation of an intact endothelial barrier (Fig. 3f). Accumulation of VE-cadherin was slightly decreased in cells incubated with Control-EVs and TGF-β-EVs and was accompanied by an increase in the number of SM22α-positive cells. SM22α staining was most prominent in cells that lost cell-cell adhesion (Fig. 3f). Notably, incubation with EVs induced the formation of gaps in HUAEC monolayer with the strongest effect exerted by TGF-β-EVs (Fig. 3f).

**Fig. 4:**
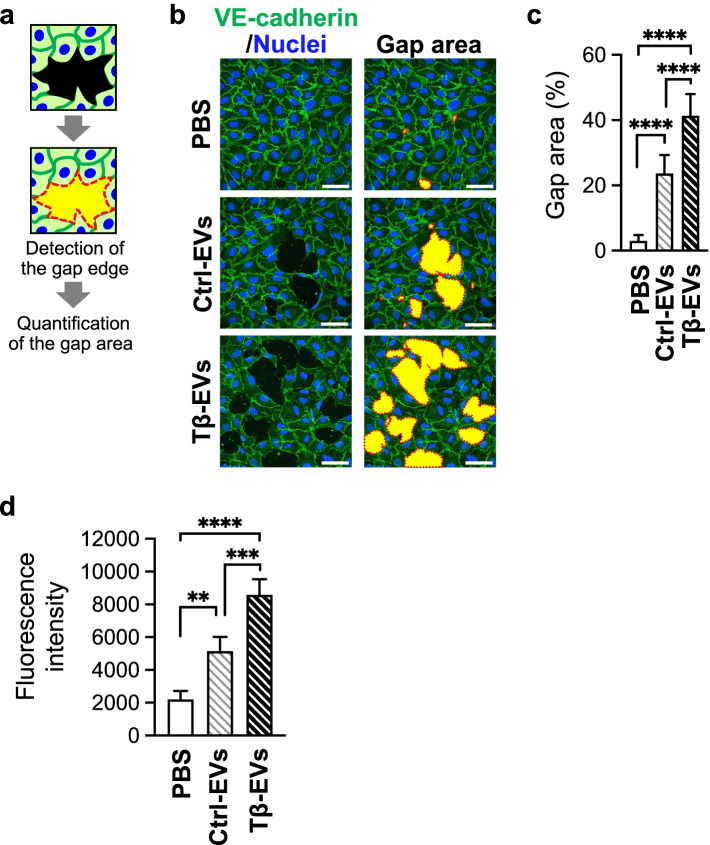
Oral cancer cell-derived EVs induce vascular destabilization. **a** Analysis of the gap area in HUAEC monolayer staining images. The gap area was determined by detecting the black (unstained) area in the cell layers and defined by the boundaries between black (unstained area) and VE-cadherin-positive area. **b** HUAEC monolayers cultured on cover slip were treated with vehicle (PBS) or EVs (Ctrl-EVs or Tβ-EVs) for 72 h. Confocal images showing the localization of VE-cadherin in HUAECs. Cells were fixed and stained with anti-VE-cadherin (green) and Hoechst33342 (nuclei: blue). The red dot line indicates the gap edge, and the yellow filled-in area indicates the gap area. Scale bars, 50 μm. **c** Quantification of the gap area. The gaps were quantified in five fields of view from at least four independent samples and represented as the percentage of formed gaps in the total area. **d** HUAEC monolayers cultured on transwell with 1-μm pore were treated with vehicle (PBS) or EVs (Ctrl-EVs or Tβ-EVs) for 72 h. Analysis of the vascular destabilization as the increase in HUAEC permeability. Permeability was analyzed by measurement of FITC-dextran (70 kDa) leakage into the lower well. Data are represented as the mean ± SD from three independent experiments. **P < 0.01, ***P < 0.001, ****P < 0.0001 by ordinary one-way ANOVA with Tukey’s multiple comparisons test

As the formed gaps corresponded to the loss of cell-cell adhesion and thus endothelial barrier integrity, we measured the area of the gaps formed in HUAEC monolayer (Fig. 4a). The presence of EVs led to the formation of significantly larger gaps compared with the control, PBS-treated HUAEC monolayer. The most significant effect was observed upon incubation with TGF-β-EVs (Fig. 4b, c) which significantly reduced cell-cell adhesion. These results were also confirmed using SAS cell-derived EVs as treatment of HUAECs with these EVs resulted in formation of gaps (Supplementary Fig. 3a, b). Moreover, gap formation frequently occurred in the vicinity of SM22α-positive cells (Fig. 3f and Supplementary Fig. 2e). In endothelial cells that have undergone EndoMT, contractility increases with the expression of SM22α [32]. Simultaneously, endothelial cells that have become contractile disrupt their cell-cell adhesions and begin to move [33]. Disruption of endothelial cell-cell adhesion is closely related to vascular destabilization by inducing increased vascular permeability. As predicted, oral cancer cell-derived EVs increased permeability of the endothelial barrier leading to vascular destabilization. This effect was more pronounced for TGF-β-EVs, which had a stronger effect on inducing the appearance of SM22α-positive cells than Control-EVs (Fig. 4d) suggesting that oral cancer cell-derived EVs induce EndoMT and gap formation in vascular endothelial monolayer. These results suggest that EndoMT is more profound in the presence of EVs derived from oral cancer cells that undergo TGF-β-induced EMT, and plays a role in the destabilization of the endothelial barrier.

## Discussion

**Fig. 5:**
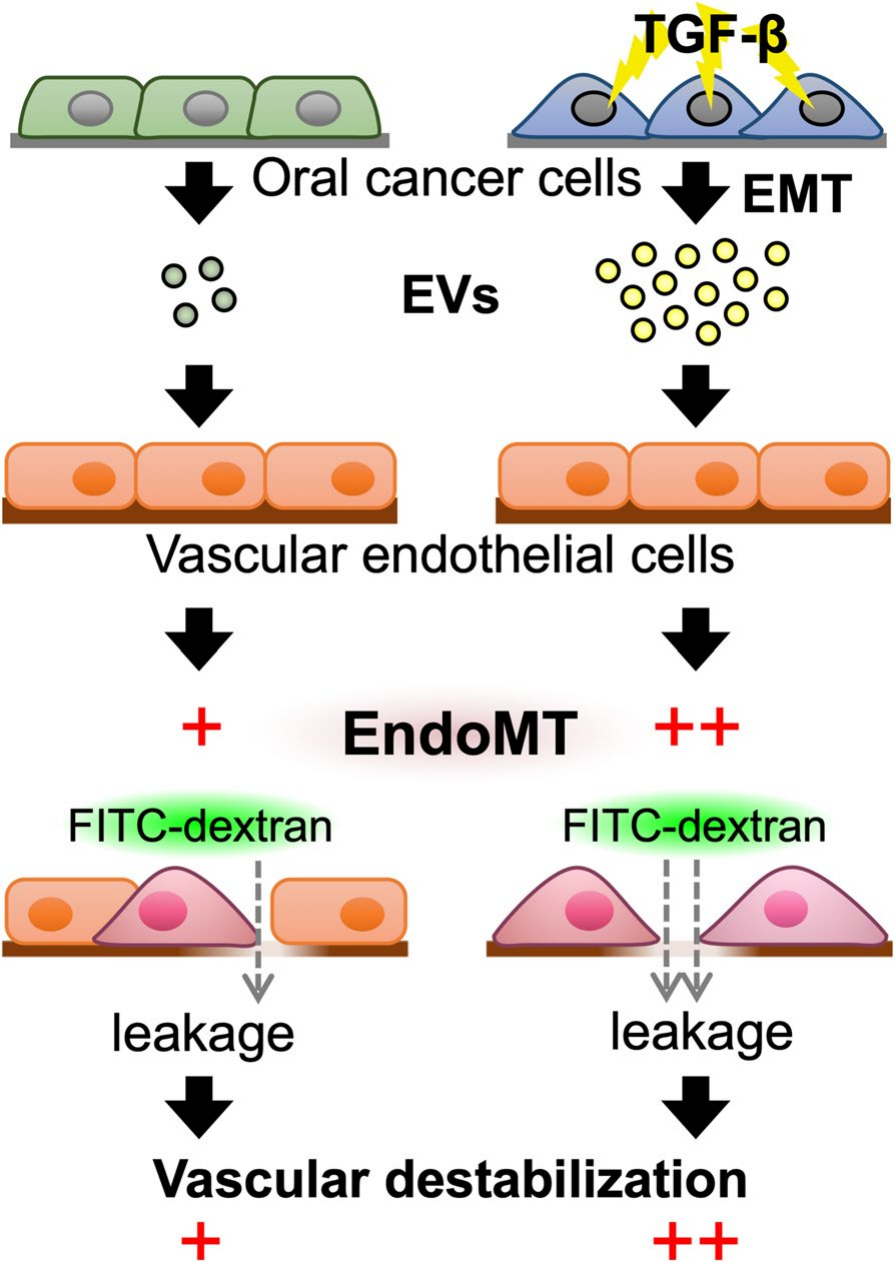
Model of EndoMT and vascular destabilization induced by oral cancer cell-derived EVs. Oral cancer cells that undergo TGF-β-induced EMT increase the release of EVs. These TGF-β-stimulated cell-derived EVs induce EndoMT in vascular endothelial cells, which leads to decreased intercellular adhesion in vascular endothelial cells and increased vascular permeability. Hence, oral cancer cells that have undergone TGF-β-induced EMT may contribute to cancer metastasis by enhancing EndoMT-associated vascular destabilization in vascular endothelial cells via EVs

In the present study, we showed that oral cancer cells in response to TGF-β increase the number of EVs which can target vascular endothelial cells. Our data also revealed that oral cancer cell-derived EVs induce EndoMT, leading to the loss of the endothelial barrier, which likely results in vascular destabilization (Fig. 5).

Metastasis is the main cause of poor prognosis in patients with OSCC. The migration of OSCC cells from their primary site can occur through the lymphatic system or blood vessels, but distant metastases of OSCC are thought to be hematogenous [34]. Notably, the presence of circulating tumor cells in the peripheral blood of patients with OSCC has been significantly associated with distant metastases. As hematogenous metastasis requires intravascular invasion and extravasation of cancer cells, disruption of cell-cell adhesion between vascular endothelial cells leading to reduction of vessel barrier function would facilitate intravasation and extravasation of cancer cells into blood vessels. Tominaga et al. demonstrated that EVs originating from brain metastatic breast cancer cells trigger the destruction of the blood-brain barrier (BBB) and promote the extravasation of cancer cells through the BBB in in vitro and in vivo models. EVs secreted by brain metastatic cancer cells altered the localization of tight junction proteins and induced actin reorganization, destroying the BBB [35]. Another group also reported that EVs containing miR-105 released from metastatic breast cancer cells destroyed the vascular endothelial barrier by inhibiting the expression of tight junction proteins, zonula occludens-1, occludin, and VE-cadherin [20]. In contrast, we observed a decrease in the accumulation of VE-cadherin at the cell-cell contact site, suggesting that the observed effect is likely independent of miR-105 action.

The role of cancer cell-derived EVs in PMN formation has received much attention. In “seed and soil theory,” EVs secreted by tumor cells enter the bloodstream and travel to distant organs where they perform local changes that favor cancer cell survival [36]. Hoshino et al. revealed that integrins present on EVs originating from metastatic breast cancer cells determine organotropic metastases of cancer cells through the formation of PMNs. The presence of integrin β3 on the surface of EVs directed EVs to the brain and facilitated uptake by brain endothelial cells [37]. Tumor-derived EVs can also target lung endothelial cells, the primary target for hematogenous metastasis of oral cancer, which has an extremely poor prognosis [34, 38]. Previous reports have shown that lung endothelial cells are targets for EVs in melanoma, breast, and colorectal cancer [39–41]. EVs from hepatocellular carcinoma can facilitate the formation of PMN and thus colonization of tumor cells by enhancing angiogenesis and inducing the permeability of pulmonary endothelial cells [42]. Additionally, EVs are known to transport their cargos to distant locations and play a role as communication tools with distant organs [13]. In distant metastasis, the vascular system acts as migration route for cancer cells. Therefore, in the step of metastatic lesion formation in distant area, vascular integrity must be destabilized to promote the extravasation of cancer cells from vessels. Oral cancer cell-derived EVs induced gap formation, leading to vascular destabilization in HUAEC monolayers (Fig. 4). Such destabilization increased permeability of normal endothelial monolayers, suggesting that EVs may also play an important role in the formation of PMN.

In our study, we observed quantitative changes in the number of EVs secreted by HSC-4 cells treated with TGF-β (Fig. 2a, b). An increased number of EVs might be important because hypoxia-dependent increased secretion of EVs from multiple myeloma cells has been shown to promote angiogenesis and contribute to cancer progression [43]. Currently, the mechanism underlying the upregulated release of EVs from oral cancer cells stimulated with TGF-β is unknown. The literature suggested that cross-talk between discoidin domain receptor-1 and TGF-β pathways regulates the release of calcifying EVs by smooth muscle cells during vascular calcification process, which is related to atherosclerosis [44]. Therefore, the activation of TGF-β signaling in oral cancer cells may also control the release of EVs. In addition, Hoshino et al. showed that enhanced EV secretion is related to invadopodia formation [45]. As TGF-β promotes the invasion of cancer cells through the formation of invadopodia [46], induction of EMT and cytoskeletal reorganization may result in increased secretion of EVs.

Biologically active molecules, namely, miRNAs and proteins packed inside EVs, are responsible for functional changes induced by EVs in recipient cells [47]. Some studies have focused on qualitative changes related to EV cargo. We also observed qualitative changes in the cargo of EVs released by HSC-4 cells treated with TGF-β when compared with EVs isolated from untreated cells (Fig. 2c). Garnier et al. revealed qualitative changes in the proteome of EVs released by human squamous carcinoma cells undergoing mesenchymal transition [48]. Proteome analysis showed enrichment of integrins and proteins involved in pathways controlling cell growth and cell motility. Another study has revealed that EVs released by mesenchymal-like breast cancer cells are characterized by the elevated expression of angiogenic factors [49]. We observed a significant increase in the gap area and permeability after EV treatment, with a more profound effect induced by TGF-β-EVs than Control-EVs (Fig. 4 and Supplementary Fig. 3). As the amount of EVs used to stimulate endothelial cells was equal, the effects induced by either Control-EVs or TGF-β-EVs are probably due to qualitative differences in the molecules contained in the two types of EVs. Yamada et al. reported that EVs derived from colon cancer cells induced partial EndoMT in human umbilical vein endothelial cells via miR-92a-3p [50].

In this study, we focused on the physiological effect of EVs on one type of endothelial cells, HUAECs. Whether oral cancer cell-derived EVs exert similar effect on other types of endothelial cells should be elucidated in the future. Further studies involving other types of endothelial cells and in vivo studies, including the molecular mechanisms leading to the phenomenon we identified, will reveal the precise role of oral cancer cell-derived EVs in the vascular microenvironment. In addition, a better understanding of the mechanisms underlying EV-mediated vascular instability will allow the development of agents targeting hematogenous metastatic events.

## Conclusion

This study revealed that oral cancer cells that underwent EMT in response to TGF-β had increased secretion of EVs. Such oral cancer cell-derived EVs induce EndoMT in vascular endothelial cells resulting in vascular destabilization. Elucidation of the molecular mechanisms underlying this phenomenon will lead to the development of novel therapeutic agents that target the formation of PMNs mediated by cancer cell-derived EVs.

## Supplementary Information

**Additional file 1: Supplementary Table 1.** List of gene-specific primers used for qRT-PCR.

**Additional file 2: Supplementary Fig. 1.** HSC-4 oral cancer cell-derived EVs induce EndoMT in vascular endothelial cells. **Supplementary Fig. 2.** SAS oral cancer cell-derived EVs induce EndoMT in vascular endothelial cells. **Supplementary Fig. 3.** SAS oral cancer cell-derived EVs induce vascular destabilization.

**Additional file 3.** Original full-length blot images for Fig. 2c.

## Data Availability

The datasets generated in the current study are available from the corresponding author on reasonable request.

## Abbreviations

BBB: Blood-brain barrier
DMEM: Dulbecco’s modified Eagle’s medium
EndoMT: Endothelial-mesenchymal transition
EMT: Epithelial-mesenchymal transition
EVs: Extracellular vesicles
FGF2: Fibroblast growth factor 2
GORASP1: Golgi reassembly-stacking protein 1
HUAECs: Human umbilical artery endothelial cells
MMP2: Matrix metalloproteinase 2
NTA: Nanoparticle tracking analysis
OSCC: Oral squamous cell carcinoma
PBS: Phosphate-buffered saline
PMN: Pre-metastatic niche
αSMA: α-Smooth muscle actin
SM22α: Smooth muscle protein 22α
TGF-β: Transforming growth factor-β
TME: Tumor microenvironment
TMEPAI: Transmembrane prostate androgen-induced protein
TSG101: Tumor susceptibility gene 101 protein
VEGFR2: Vascular endothelial growth factor receptor 2
