# Supplementary material for: Transforming growth factor-β-induced secretion of extracellular vesicles from oral cancer cells evokes endothelial barrier instability via endothelial-mesenchymal transition
Source: Inflamm Regen. 2022 Sep 4;42:38. doi: 10.1186/s41232-022-00225-7 (PMC9441046; doi:10.1186/s41232-022-00225-7)
Supplement: Supplementary file 1 — Additional file 1: Supplementary Table 1. List of gene-specific primers used for qRT-PCR. [file 41232_2022_225_MOESM1_ESM.pdf]

Supplementary Table 1. List of gene-specific primers used for qRT-PCR

Transcript	Forward (F)/ Reverse (R)	sequence（5’→ 3’）
β-actin	F	TCACCCACACTGTGCCCATCTACGA
	R	CAGCGGAACCGCTCATTGCCAATGG
Claudin-1	F	CTGGGAGGTGCCCTACTTTG
	R	ACACGTAGTCTTTCCCGCTG
E-cadherin	F	TGCACCAACCCTCATGAGTG
	R	GTCAGTATCAGCCGCTTTCAG
FGF2	F	GCGGCGTCACATCTTCTACA
	R	CCCAGAAAACCCGAGCGA
MMP2	F	TACTGAGTGGCCGTGTTTGC
	R	GGGAGCAGAGATTCGGCTTT
αSMA	F	TGAGCGTGGCTATTCCTTCGT
	R	GCAGTGGCCATCTCATTTTCAA
SM22α	F	TCAAGCAGATGGAGCAGGTG
	R	GCTGCCATGTCTTTGCCTTC
Slug	F	GCCTCCAAAAAGCCAAACTACA
	R	GAGGATCTCTGGTTGTGGTATGACA
Snail	F	TTCTCACTGCCATGGAATTCC
	R	GCAGAGGACACAGAACCAGAAA
Tie2	F	GGTGGAAAAGCCCTTCAACA
	R	CATCCCCAAAGTAAGGCTCAG
TMEPAI	F	TTTGTGGGAATGGCTTTGCG
	R	GAGGACACTGGGCTTCAACA
VEGFR2	F	CAGAATCCCTGCGAAGTACCTT
	R	GTCAGTACATGCCCCGCTTTAA
Vimentin	F	GCAAAGATTCCACTTTGCGT
	R	GAAATTGCAGGAGGAGATGC
