# Supplementary material for: Transforming growth factor-β-induced secretion of extracellular vesicles from oral cancer cells evokes endothelial barrier instability via endothelial-mesenchymal transition
Source: Inflamm Regen. 2022 Sep 4;42:38. doi: 10.1186/s41232-022-00225-7 (PMC9441046; doi:10.1186/s41232-022-00225-7)
Supplement: Supplementary file 2 — Additional file 2: Supplementary Fig. 1. HSC-4 oral cancer cell-derived EVs induce EndoMT in vascular endothelial cells. Supplementary Fig. 2. SAS oral cancer cell-derived EVs induce EndoMT in vascular endothelial cells. Supplementary Fig. 3. SAS oral cancer cell-derived EVs induce vascular destabilization. [file 41232_2022_225_MOESM2_ESM.pdf]

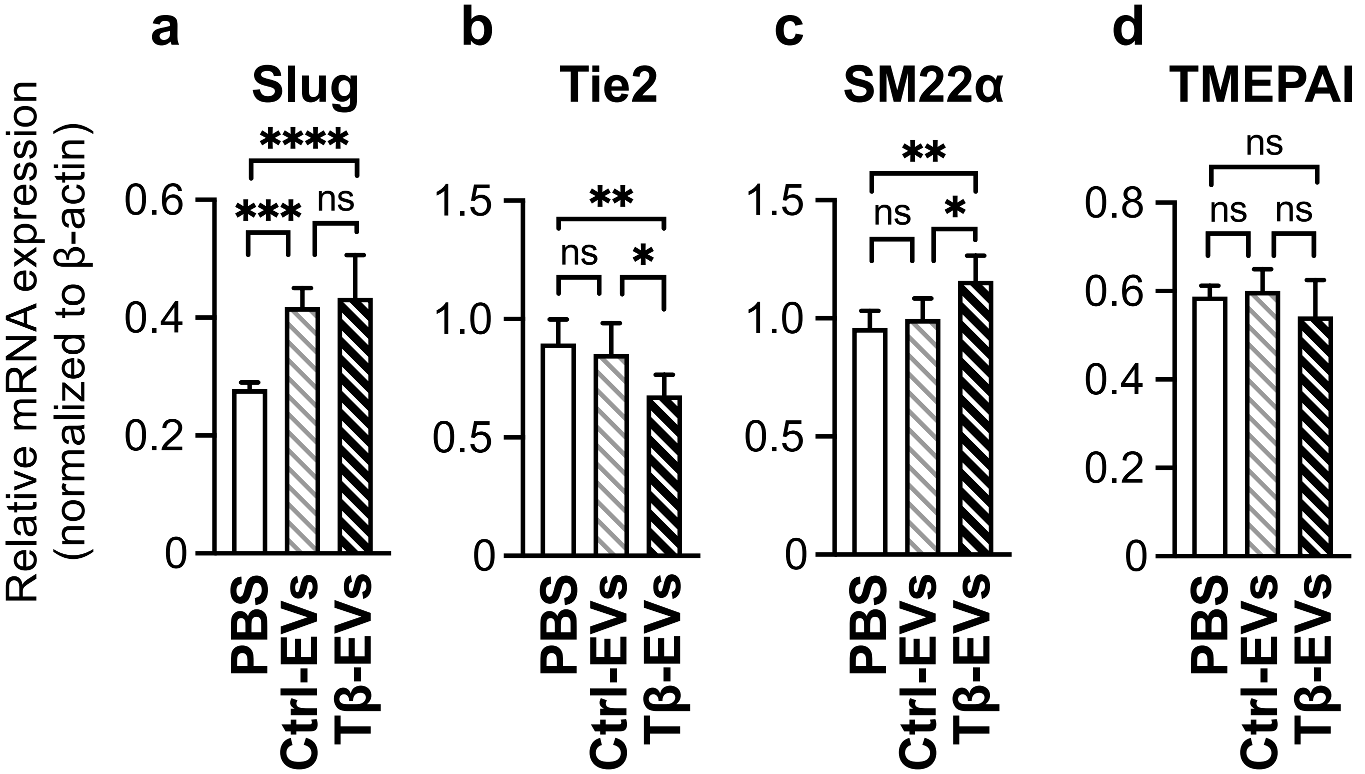

Supplementary Fig. 1 HSC-4 oral cancer cell-derived EVs induce EndoMT in vascular endothelial cells. a-d HUAEC monolayers were treated with vehicle (PBS) or EVs (Ctrl-EVs or Tβ-EVs) for 72 h. The expression of Slug (a), Tie2 (b), SM22α (c) or TMEPAI (d) was analyzed by qRT-PCR. Data represent fold changes relative to β-actin levels. All data are shown as mean ± SD from three independent experiments. **P* < 0.05, ***P* < 0.01, ****P* < 0.001, *****P* < 0.0001 by ordinary one-way ANOVA with Tukey's multiple comparisons test; ns, not significant.

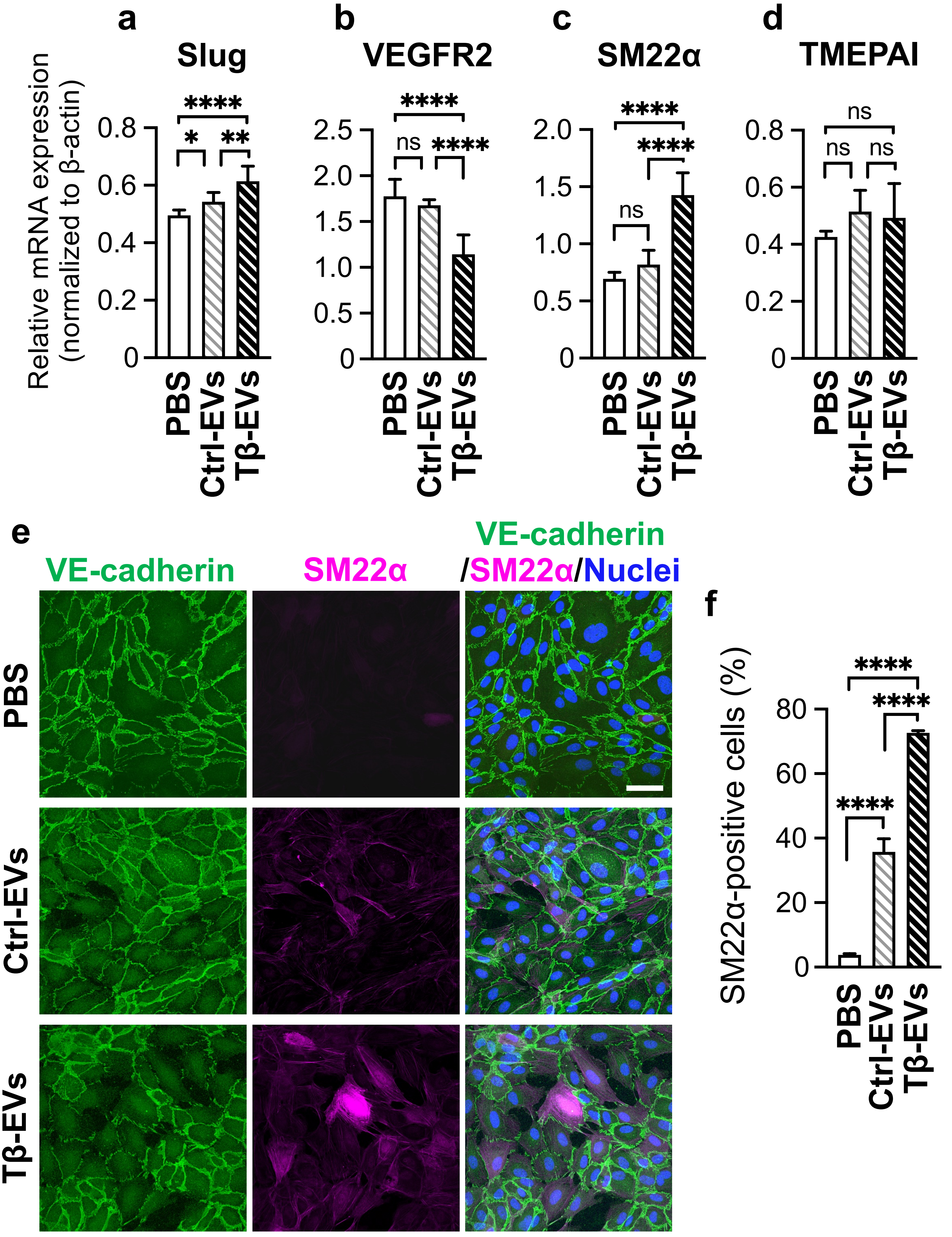

Supplementary Fig. 2. See next page for caption.

Supplementary Fig. 2 SAS oral cancer cell-derived EVs induce EndoMT in vascular endothelial cells. HUAEC monolayers were treated with vehicle (PBS) or EVs (Ctrl-EVs or Tβ-EVs) for 72 h. **a-d** The expression of Slug (**a**), VEGFR2 (**b**), SM22α (**c**) or TMEPAI (**d**) was analyzed by qRT-PCR. Data represent fold changes relative to β-actin. **e** Confocal images showing both the localization of VE-cadherin and the expression of SM22α in HUAECs. Cells were fixed and stained with anti-VE-cadherin (green) and anti-SM22α (magenta) antibodies. Nuclei were stained with Hoechst33342 (blue). Scale bars, 50 μm. **f** Quantification of SM22α-positive cells. All data are shown as the mean ± SD from three independent experiments. *$P < 0.05$, **$P < 0.01$, ****$P < 0.0001$ by ordinary one-way ANOVA with Tukey's multiple comparisons test; ns, not significant.

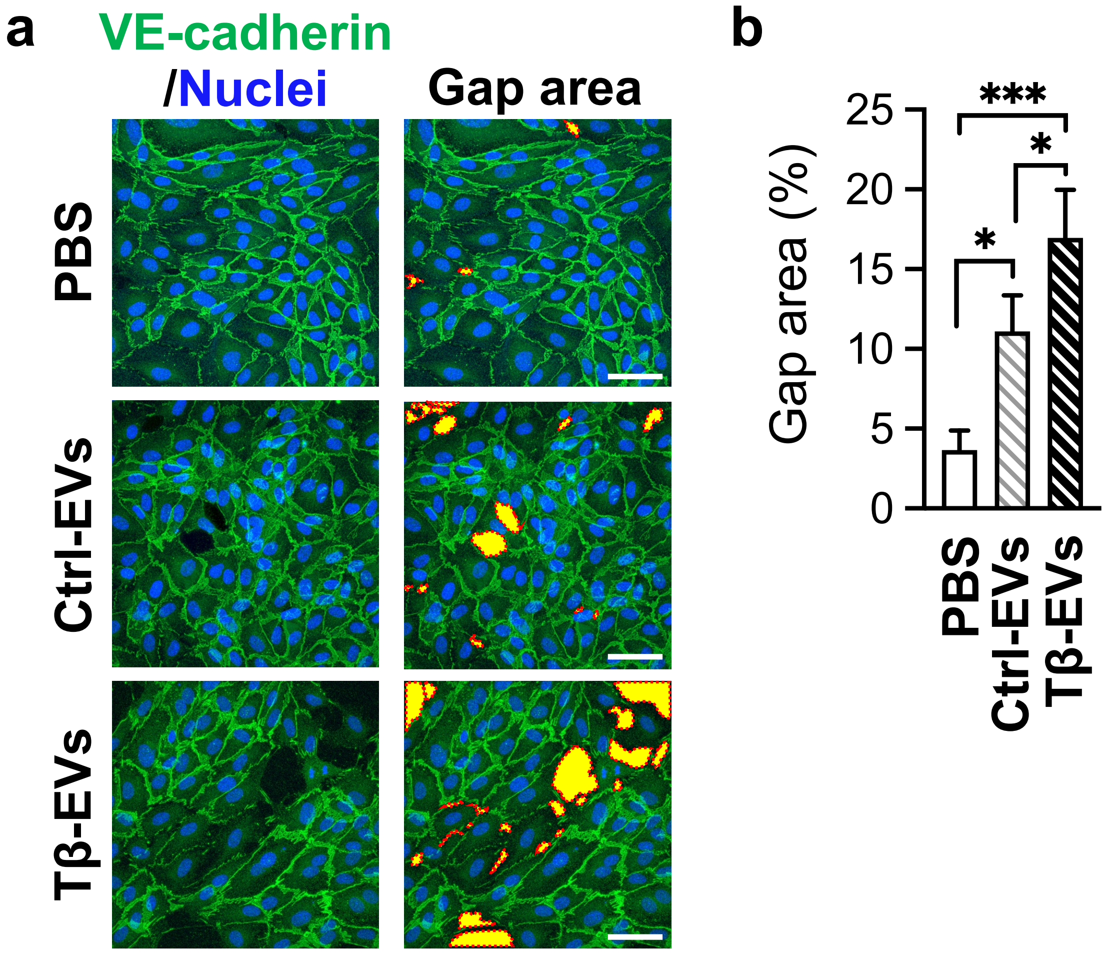

Supplementary Fig. 3 SAS oral cancer cell-derived EVs induce vascular destabilization. HUAEC monolayers cultured on cover slip were treated with vehicle (PBS) or EVs (Ctrl-EVs or Tβ-EVs) for 72 h. **a** Gap area in HUAECs monolayer staining images. See Fig. 4 for the determination of gap area. Confocal images showing the localization of VE-cadherin in HUAECs. Cells were fixed and stained with anti-VE-cadherin (green) and Hoechst33342 (nuclei: blue). The red dot line indicates the gap edge, and the yellow filled-in area indicates the gap area. Scale bars, 50 μm. **b** Quantification of gap area. The gaps were quantified in five fields of view from at least four independent samples. All data are shown as the mean ± SD from three independent experiments. *$P < 0.05$, *** $P < 0.001$ by ordinary one-way ANOVA with Tukey's multiple comparisons test; ns, not significant.
