# Supplementary material for: Transforming growth factor-β-induced secretion of extracellular vesicles from oral cancer cells evokes endothelial barrier instability via endothelial-mesenchymal transition
Source: Inflamm Regen. 2022 Sep 4;42:38. doi: 10.1186/s41232-022-00225-7 (PMC9441046; doi:10.1186/s41232-022-00225-7)
Supplement: Supplementary file 3 — Additional file 3. Original full-length blot images for Fig. 2c. [file 41232_2022_225_MOESM3_ESM.pdf]

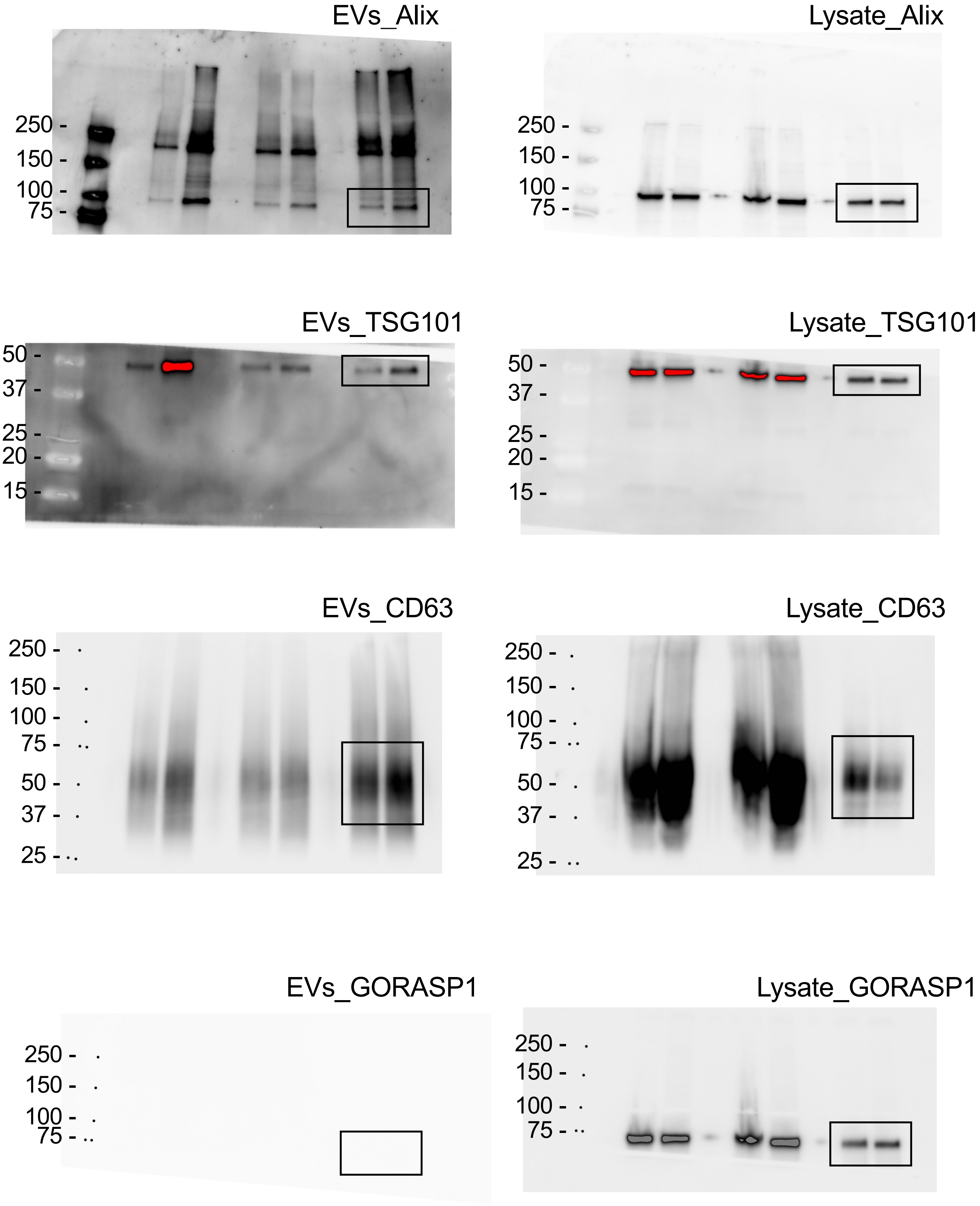

Fig. 2c Original full-length blot images: Kobayashi et al.
